# Earthworm (Oligochaeta, Lumbricidae) intraspecific genetic variation and polyploidy

**DOI:** 10.18699/vjgb-24-62

**Published:** 2024-09

**Authors:** S.V. Shekhovtsov, Ye.A. Derzhinsky, E.V. Golovanova

**Affiliations:** Institute of Cytology and Genetics of the Siberian Branch of the Russian Academy of Sciences, Novosibirsk, Russia Institute of Biological Problems of the North of the Far-Eastern Branch of the Russian Academy of Sciences, Magadan, Russia; Vitebsk State University named after P.M. Masherov, Vitebsk, Belarus; Omsk State Pedagogical University, Omsk, Russia

**Keywords:** genetic lineages, karyotypes, phylogeny, species divergence, генетические линии, кариотипы, филогения, дивергенция видов

## Abstract

Earthworms are known for their intricate systematics and a diverse range of reproduction modes, including outcrossing, self-fertilization, parthenogenesis, and some other modes, which can occasionally coexist in a single species. Moreover, they exhibit considerable intraspecific karyotype diversity, with ploidy levels varying from di- to decaploid, as well as high genetic variation. In some cases, a single species may exhibit significant morphological variation, contain several races of different ploidy, and harbor multiple genetic lineages that display significant divergence in both nuclear and mitochondrial DNA. However, the relationship between ploidy races and genetic lineages in earthworms remains largely unexplored. To address this question, we conducted a comprehensive review of available data on earthworm genetic diversity and karyotypes. Our analysis revealed that in many cases, a single genetic lineage appears to encompass populations with different ploidy levels, indicating recent polyploidization. On the other hand, some other cases like Octolasion tyrtaeum and Dendrobaena schmidti/D. tellermanica demonstrate pronounced genetic boundaries between ploidy races, implying that they diverged long ago. Certain cases like the Eisenia nordenskioldi complex represent a complex picture with ancient divergence between lineages and both ancient and recent polyploidization. The comparison of phylogenetic and cytological data suggests that some ploidy races have arisen independently multiple times, which supports the early findings by T.S. Vsevolodova-Perel and T.V. Malinina. The key to such a complex picture is probably the plasticity of reproductive modes in earthworms, which encompass diverse modes of sexual and asexual reproduction; also, it has been demonstrated that even high-ploidy forms can retain amphimixis.

## Abstract (ru)

Дождевые черви известны своей сложной систематикой и разнообразным набором типов размножения, включая амфимиксис, самооплодотворение, партеногенез и некоторые другие способы, которые иногда могут сосуществовать в пределах одного вида. Более того, они демонстрируют значительное внутривидовое разнообразие кариотипов с уровнями плоидности от ди- до декаплоидных и выше, а также высокую генетическую изменчивость. В некоторых случаях один вид может сочетать большую морфологическую изменчивость, несколько рас с разной плоидностью и несколько филогенетических линий со значительными различиями как по ядерной, так и по митохондриальной ДНК. При этом соответствие между расами различной плоидности и генетическими линиями дождевых червей остается в значительной степени неисследованным. В связи с этим мы провели всесторонний обзор имеющихся данных о генетическом разнообразии и кариотипах дождевых червей. Наш анализ показал, что во многих случаях одна генетическая линия включает в себя популяции с разными уровнями плоидности, что указывает на недавнюю полиплоидизацию. С другой стороны, в некоторых случаях, как, например, Octolasion tyrtaeum и Dendrobaena schmidti/D. tellermanica, имеют место выраженные генетические границы между расами, что означает давнюю дивергенцию между ними. Некоторые таксоны, такие как комплекс Eisenia nordenskioldi, представляют собой множество давно дивергировавших филогенетических линий сo сложными родственными отношениями между ними и как древней, так и недавней полиплоидией. Сопоставление филогенетических и цитологических данных позволяет предположить, что некоторые полиплоидные расы независимо возникали несколько раз, что подтверждает выводы работы Т.С. Всеволодовой-Перель и Т.В. Малининой. Причиной такой сложной картины, вероятно, служит пластичность способов репродукции, которые включают в себя разнообразные виды полового и бесполого размножения; причем даже формы с высокой плоидностью могут размножаться амфимиктически.

## Introduction

Polyploidy in animals is relatively rare (Muller, 1925; Orr, 1990). However, certain groups are exceptions to this rule and exhibit a significant incidence of polyploidy (Gregory, Mable, 2005). Earthworms are among these exceptions (Muldal, 1952; Viktorov, 1997): the initial studies demonstrated that polyploidy is observed not only among groups of closely related species, but even within a single species, and often in sympatry (Omodeo, 1952, 1955). Subsequently, this phenomenon was documented in representatives of diverse genera (Vsevolodova-Perel, Bulatova, 2008; Mezhzherin et al., 2018). In addition to that, earthworms demonstrate diverse ways of reproduction (Pavlíček et al., 2023). Although in animals polyploidy is generally associated with parthenogenesis, polyploid earthworms often retain the ability for amphimixis (Viktorov, 1989). While some species comprise a set of races with different ploidy levels, the prevailing view is that this alone is not a sufficient reason to classify them as distinct species (Vsevolodova-Perel, Bulatova, 2008).

Molecular studies revealed a vast genetic diversity within earthworm species (King et al., 2008; Porco et al., 2013). In most cases, several well-defined clades within a given species were identified, with 15–20 % of nucleotide substitutions between mitochondrial genes (these estimates sometimes vary, because different studies employ various distance measures, like Kimura-2-parameter, etc.). These clades are commonly referred to either as cryptic species or as the so-called genetic lineages (Marchán et al., 2018). The attempts to clarify the issue of genetic divergence on the nuclear level using various molecular methods generally confirmed the existence of significant nucleotide distances between these lineages (Martinsson, Erséus, 2017; Taheri et al., 2018), although in some instances, the data did not demonstrate signs of reproductive isolation of distinct lineages differing on the mitochondrial level (Giska et al., 2015; Martinsson et al., 2017; Martinsson, Erséus, 2018).

Thus, we can see that certain earthworm species have multiple races with different ploidy levels, as well as several genetic lineages with distinct mitochondrial and nuclear genomes. However, the relationship between chromosomal and DNA sequence variation remains unclear. Does each chromosomal race correspond to a particular genetic lineage, or do the boundaries between these entities lie elsewhere?

In this review, we analyzed the patterns of chromosomal and molecular variation in several earthworm species from various genera within the family Lumbricidae. The results provide insight into the relationships between these entities and outline directions for future research.

## Materials and methods

The data on the chromosome numbers of the populations of various earthworm species were taken from published materials (Muldal, 1952; Omodeo, 1952, 1955; Vedovini, 1973; Graphodatsky et al., 1982; Bulatova et al., 1984, 1987; Perel, Graphodatsky, 1984; Casellato, 1987; Viktorov, 1989, 1997; Kashmenskaya, Polyakov, 2008; Vsevolodova-Perel, Bulatova, 2008; Vlasenko et al., 2011; Mezhzherin et al., 2018). The information on the number of genetic lineages and on the assignment of particular populations to genetic lineages was extracted from scientific papers (Heethoff et al., 2004; King et al., 2008; Porco et al., 2013; Fernández et al., 2016; Shekhovtsov et al., 2014, 2020a–d; Ermolov et al., 2023), as well as the GenBank database.

**Fig. 1.**
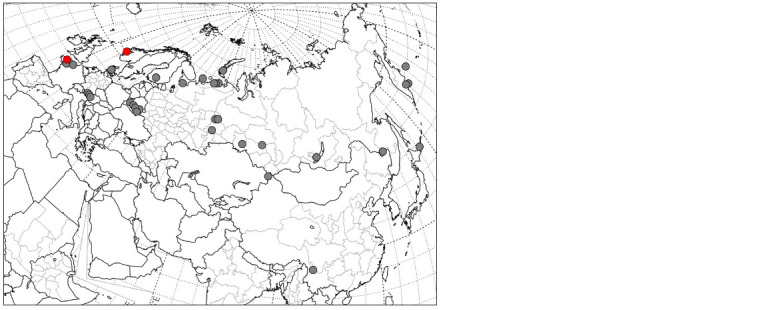
Sampling locations of the sequenced D. octaedra individuals from Eurasia. Russia, Belarus, and Kazakhstan, our data; other countries, GenBank. Grey dots – lineage 1; red dots – lineage 2.

For Dendrobaena octaedra (Savigny, 1826), we also obtained a sequence dataset for 99 specimens from 24 populations from Russia and adjacent countries (Fig. 1). Briefly, earthworms were fixed in ethanol; DNA was extracted from whole individuals or from parts of the body (ca. 100 mg) using BioSilica columns (Dia-m, Russia) according to the manufacturers’ instructions. Fragments of the cox1 gene were amplified using universal primers and sequenced as described in (Shekhovtsov et al., 2013). Sequences were deposited in GenBank under accession numbers OR366494–OR366522, KJ772497, KJ772504, KX400644, MH755642, MH755644, MH755645, MH755647, MH755649, MH755654, MH755666, MH755670, MH755672. A dataset of 157 full-length 658 bp cox1 barcodes was taken from GenBank. Unique haplotypes were extracted from these datasets. Sequences of D. octaedra L2 were additionally searched in the BOLD database (https://v4.boldsystems.org/). Maximum likelihood trees were constructed using RAxML v. v. 8.2.12 (Stamatakis, 2014) with the GTRCAT substitution model and 1000 bootstrap replicates. Bayesian analysis was performed in MrBayes v. 3.4 (Ronquist et al., 2012). Two simultaneous independent runs were performed with 10 million generations each; 25 % of the generations were discarded as burn-in.

## Results

Dendrobaena octaedra

**Fig. 2.**
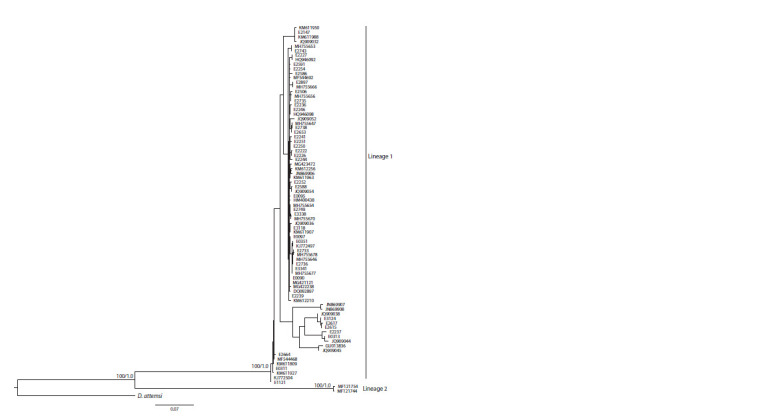
The phylogenetic tree built for the D. octaedra haplotypes using the maximum likelihood method. Numbers near branches indicate bootstrap support/Bayesian posterior probabilities.

Among the 99 D. octaedra sequences obtained by us, we found 40 unique haplotypes. We also extracted 157 sequences from GenBank with 41 unique haplotypes. We combined these unique haplotypes from the two samples to construct phylogenetic trees (Fig. 2). Our analysis revealed that average genetic diversity within D. octaedra is very low compared to other earthworms. The majority of haplotypes belonged to a single group with lower diversity: average p-distance within the group was 2.3 %.

However, two haplotypes from GenBank, MF121744 and MF121754, differed significantly from the rest of the sample with an average p-distance of 19 %. These specimens were designated by the authors as Dendrobaena octaedra complex sp. L2. They were collected in the Eawy forest in Normandy (France), near the English Channel. Another three closely related sequences were found in the BOLD database, one from the vicinities of Florelandet (Norway), and the collection points of the other two specimens were undisclosed. These regions were affected by the most recent glaciation, so the local populations of D. octaedra were obviously introduced from another region relatively recently (ca. 10 kya). There are too few data on these specimens; since there are no associated papers with morphological descriptions, there is a chance that they might belong to another yet unknown species.

Four chromosomal races are known within D. octaedra, 4n = 72, 5n = 90, 6n = 108, and 8n = 144 (Casellato, 1987; Viktorov, 1993; Mezhzherin et al., 2018). Since the predominant majority of D. octaedra populations belong to a single genetic lineage, we can suggest that these three chromosomal races coexist within this lineage.

Aporrectodea rosea (Savigny, 1826)

Races with 2n = 36, 3n = 54, 4n = 72, 5n = 90, 6n = 108, 8n = 144, and 10n = ~180 were described for A. rosea (Muldal, 1952; Casellato, Rodighiero, 1972; Casellato, 1987; Vsevolodova-Perel, Bulatova, 2008; Vlasenko et al., 2011). The initial barcoding studied uncovered the existence of several genetic lineages within this species: R.A. King et al. (2008) detected three lineages, whereas D. Porco et al. (2013) discovered four. R. Fernández et al. (2016) performed a detailed phylogeographic analysis of A. rosea in Western Europe, demonstrating that it can be divided into two major clades: the Eurosiberian and the Mediterranean. The former has a cosmopolitan distribution and includes the four lineages identified by R.A. King et al. (2008) and D. Porco et al. (2013), while the latter is confined to the Mediterranean region. Subsequently, additional genetic lineages were found in Russia and adjacent countries, all belonging to the Eurosiberian clade (Shekhovtsov et al., 2020a).

Therefore, many genetic lineages and ploidy races coexist within A. rosea. Detailed data on the relationships between them are currently not available. However, there is a single example that can shed light on this issue: S.V. Mezhzherin et al. (2018) reported a case of four chromosomal races (2n, 3n, 6n, and 8n) in the A.V. Fomin Botanical Garden (Kyiv). According to phylogeographic studies (King et al., 2008; Shekhovtsov et al., 2020a), three lineages are rarely found in sympatry, and four have never been reported. Therefore, it is plausible that in this case, several chromosomal races coexist within a single lineage. Sure, this cannot be called hard evidence, but we don’t have better data so far.

It is worth noting that body size does not correlate with chromosome number in A. rosea (Vlasenko et al., 2011). It is presumed that the races with 2n = 36, as well as at least some populations with 4n = 72 and 6n = 108 are amphimictic (Vsevolodova-Perel, Bulatova, 2008).

Bimastos rubidus (Eisen, 1874)

B. rubidus (formerly known as Dendrodrilus rubidus) is a rare species containing only a single genetic lineage (Ermolov et al., 2023) despite the fact that it has considerable intraspecific diversity and was until recently considered to contain four subspecies (Holmstrup, Simonsen, 1996; Vsevolodova-Perel, 1997; Sims, Gerard, 1999; Csuzdi et al., 2017). Chromosomal studies reported the presence of six ploidy races within the species: 2n = 34, 3n = 51, 4n = 68, 5n = 85, 6n = 102, and 8n = 136 (Muldal, 1952; Omodeo, 1952; Vedovini, 1973; Casellato, 1987; Mezhzherin et al., 2018). Thus, similar to D. octaedra, multiple ploidy races are encompassed within a single genetic lineage

Octolasion tyrtaeum (lacteum) (Örley, 1881

O. tyrtaeum is generally believed to comprise two discrete size groups, referred to as “small” (body length 4–8 cm) and “big” (10–14 cm) (Meinhardt, 1974; Heethoff et al., 2004). Molecular studies demonstrated that mitochondrial and nuclear gene sequences of these two groups are significantly different and belong to two distinct genetic lineages (Heethoff et al., 2004; Shekhovtsov et al., 2014). These two lineages were reported to differ in ploidy: the “small” one is diploid, while the “big” one is triploid (Mezhzherin et al., 2018). Thus, in this case we can observe that a ploidy race corresponds to a single lineage. O. tyrtaeum is also a rare example of the dependence of body size on ploidy in earthworms (Mezhzherin et al., 2018).

It should be noted that this division of O. tyrtaeum into two groups is not straightforward. A third genetic lineage with body size similar to the “small” lineage but with different body proportions was found (Shekhovtsov et al., 2014, 2020b). Its ploidy is unknown. Moreover, body size may also differ between populations of different lineages (Shekhovtsov et al., 2020b).

Dendrobaena schmidti (Michaelsen, 1907)

D. schmidti is widespread in the Caucasus and adjacent regions. It exhibits a wide range of pigmentation intensity, from unpigmented to deep purple coloration, and body size, ranging from 35 to 160 mm. Due to this variation, many subspecies were isolated from D. schmidti, some of them later recognized as distinct species (Perel, 1966; Kvavadze, 1985; Vsevolodova-Perel, 2003). However, not all these subspecies were widely accepted by researchers due to the lack of clear boundaries between them (Vsevolodova-Perel, 2003).

Chromosomal studies demonstrated that all the subspecies of D. schmidti distinguished in the book of E.S. Kvavadze (1985) exhibit the same chromosome number of 2n = 36 (Bakhtadze et al., 2003, 2005). On the other hand, D. tellermanica, originally described as D. s. tellermanica in 1966 (Perel, 1966) and subsequently elevated to the species rank (Vsevolodova-Perel, 2003), is tetraploid (4n = 72) (Bakhtadze et al., 2003, 2005). D. tellermanica was distinguished from D. schmidti based on the lack of pigmentation, the start of the clitellum on the 25th segment (vs. 26th in D. schmidti), and wider distribution beyond the Caucasus region. Initially, it was believed to be strictly parthenogenetic, but later studies revealed the presence of populations with mature spermatozoa and spermatophores (Vsevolodova-Perel, 2003).

Recent molecular studies (Shekhovtsov et al., 2020c, 2023) showed that while D. schmidti and D. tellermanica are related, they exhibit significant differences in terms of nucleotide substitutions. This implies relatively ancient polyploidization, similar to O. tyrtaeum.

Eisenia nordenskioldi (Eisen, 1879) complex

E. nordenskioldi has a vast distribution in Northern Asia and adjacent areas and is known for its high morphological diversity (Malevich, 1956; Vsevolodova-Perel, 1997). Thus it is not surprising that it was found to have extensive genetic diversity (Blakemore, 2013; Shekhovtsov et al., 2013, 2016, 2018; Hong, Csuzdi, 2016). Molecular studies revealed that E. nordenskioldi consists of multiple genetic lineages divided into two large clades (Shekhovtsov et al., 2020d). These lineages strongly differ in mitochondrial and nuclear genome sequences (Shekhovtsov et al., 2020c), as well as genome size (Shekhovtsov et al., 2021). Therefore, E. nordenskioldi should be regarded as a species complex. Preliminary, this complex was divided into two large clades, referred to as E. nordenskioldi s. str. (genetic lineages 6, 7, and 9) and Eisenia sp. 1 aff. E. nordenskioldi (all other lineages) (Shekhovtsov et al., 2020d).

E. nordenskioldi is probably the best studied model of karyotype diversity among earthworms (Graphodatsky et al., 1982; Bulatova et al., 1984, 1987; Perel, Graphodatsky, 1984; Viktorov, 1989, 1997; Kashmenskaya, Polyakov, 2008; Vsevolodova-Perel, Bulatova, 2008). Races with 2n = 36, 4n = 72, 6n = 96–102, and 8n = 142–152 were identified (Viktorov, 1997). However, it is not yet clear how the division into genetic lineages correlates with the different ploidy races. Although no direct studies are available, published data allow one to attribute certain populations of E. nordenskioldi to specific lineages and races. For example, the population from Magadan with 8n = 152 chromosomes (Viktorov, 1989) belongs to lineage 9: individuals collected from the same locations were sent to A.G. Viktorov and the authors of this paper by D.I. Berman. Furthermore, extensive studies failed to find other lineages of the pigmented form of E. nordenskioldi in this region (Shekhovtsov et al., 2020d).

M.N. Kashmenskaya and A.V. Polyakov (2008) conducted a study on the chromosome set of two individuals identified as E. n. nordenskioldi and E. atlavyniteae, a closely related species isolated from E. nordenskioldi (Perel, Graphodatsky, 1984), from the Central Siberian Botanical Garden in Novosibirsk. Both individuals were found to be diploid (2n = 36). A later study of earthworms from the same location found lineages 1, 2, and 3 of the pigmented form of E. nordenskioldi (Shekhovtsov et al., 2013). Although we cannot attribute the specimens from the study of M.N. Kashmenskaya and A.V. Polyakov (2008) to a precise lineage, we know that this location does not harbor any lineage of E. nordenskioldi s. str. Therefore, these diploid populations belong to Eisenia sp. 1 aff. E. nordenskioldi.

Tetra- and octoploid races of E. nordenskioldi were reported from the Taymyr Autonomous Okrug, located in the north of West Siberia (Bulatova et al., 1984; Viktorov, 1989; Vsevolodova-Perel, Leirikh, 2014). Molecular studies identified genetic lineages 1 and 9 of the pigmented form of E. nordenskioldi from the same region (Shekhovtsov et al., 2020d). T.V. Malinina and T.S. Perel (1984) used allozyme data to demonstrate that the octoploid population from Taymyr is related to those from the south of West Siberia compared to other regions, suggesting that it likely belongs to lineage 1.

The population of E. nordenskioldi from the Dzhanybek experimental station of the Institute of Forest Science RAS, located in the steppe zone of European Russia in Volgograd Oblast, has been reported to have 4n = 72 (Malinina, Perel, 1984; Viktorov, 1989). This population was artificially introduced from the floodplain of the Eruslan River in Saratov Oblast, Russia (Vsevolodova-Perel, Bulatova, 2008). According to our data, only lineage 7 of E. nordenskioldi is found in this region. The population from the Prioksko-Terrasny Nature Reserve in Moscow Oblast is also within the distribution range of lineage 7. Moreover, T.V. Malinina and T.S. Perel (1984) suggested that it is related to the Dzhanybek population based on allozyme data, so we could also attribute it to lineage 7. The same can be hypothesized for the Kursk population, which has 6n = 102 chromosomes (Viktorov, 1989).

A.G. Viktorov (1989) reported that E. nana from East Kazakhstan Oblast has 34 chromosomes. It has since been discovered that this species is actually a synonym of lineage 5 of the pigmented form of E. nordenskioldi (Shekhovtsov et al., 2020d; Golovanova et al., 2021). As the individuals used for both genetic and chromosomal analyses were collected from the same region, it is reasonable to hypothesize that they belong to the same lineage.

The unpigmented pallida form of E. nordenskioldi is considered to be diploid (Vsevolodova-Perel, Leirikh, 2014). These data were obtained for the population from the Novosibirsk Akademgorodok (Malinina, Perel, 1984; Viktorov, 1989). However, the pallida form is distributed throughout Siberia and the Far East and also contains many genetic lineages with different distributions (Shekhovtsov et al., 2016). The pallida form from Akademgorodok belongs to lineage 6 (Shekhovtsov et al., 2020d). It has a small genome size (ca. 270 Mb) (Shekhovtsov et al., 2021), while lineage 1 of the pallida form has a big genome (ca. 2500 Mb), suggesting that it may be polyploid

**Fig. 3.**
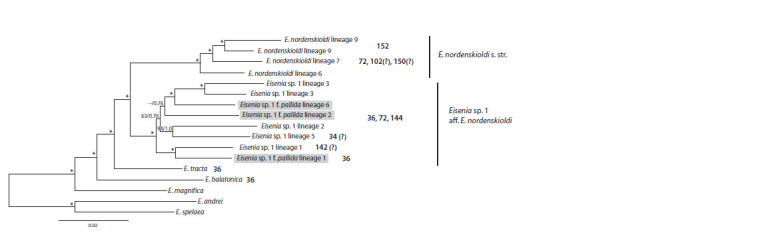
The position of chromosomal races of E. nordenskioldi on the phylogenetic tree of the species according to published data. The tree was taken from (Shekhovtsov et al., 2020d) built using transcriptomic data of various genetic lineages of E. nordenskioldi and outgroup species using the maximum likelihood algorithm. Grey boxes around lineage names indicate that these lineages belong to the pallida (unpigmented) form. Bold numbers indicate chromosome numbers; question signs indicate that karyotype assignment is tentative. Numbers near branches indicate bootstrap support/Bayesian posterior probabilities. * Refers to 100/1.0.

We summarized the obtained data in Fig. 3, which includes chromosome numbers and the phylogenetic tree constructed using 212 nuclear genes (Shekhovtsov et al., 2020d). However, it is important to note that the chromosome numbers displayed are representative of certain populations and may not apply to the entire lineage

Eisenia tracta, the sister species of the E. nordenskioldi complex, has 2n = 36 chromosomes, as does its relative E. balatonica (Fig. 3). Therefore, it is reasonable to suggest that the ancestors of the two clades of E. nordenskioldi were also diploid with 36 chromosomes, and that polyploidy arose independently in both clades. This hypothesis on the independent origin of polyploid races in E. nordenskioldi was proposed by T.V. Malinina and T.S. Perel (1984) based on allozyme data. The authors concluded that octoploid populations arose independently at least twice. Our data supports this position, as octoploid races appear to have arisen independently in the two large clades of E. nordenskioldi (Fig. 3).

It is worth noting that published papers (Viktorov, 1997; Vsevolodova-Perel, Bulatova, 2008) and our unpublished data indicate that all studied populations of E. nordenskioldi have well-developed testes and normal spermatogenesis. According to A.G. Viktorov (1997), the evidence for parthenogenesis was only observed for the acystis form from Central Asia, which was subsequently isolated into a separate species (Vsevolodova-Perel, 1997). Additionally, octoploid individuals of lineage 9 from Magadan were observed copulating (D.I. Berman, personal communication). Thus, the available evidence suggests that polyploidy does not result in the loss of sexual reproduction in the E. nordenskioldi complex in most cases

## Discussion

Based on the data presented above, it is apparent that the relationships between ploidy races and genetic lineages are rarely straightforward. This was only observed in the cases of O. tyrtaeum and the D. schmidti – D. tellermanica pair. For many species, multiple ploidy races were found to belong to the same genetic lineage. In all these cases, races of different ploidy do not have any apparent differences on the level of mitochondrial or nuclear DNA, suggesting that polyploidization events in these cases may be recent. However, in other cases, such as the E. nordenskioldi complex, the age of the polyploidization events is unknown, but is likely to be significant, 1–3 Mya as estimated in (Shekhovtsov et al., 2013). Although the precision of molecular clock dating using only mitochondrial data and no fossils is limited (Kodandaramaiah, 2011), deep divergence between these taxa is obvious

Earthworms exhibit a high degree of plasticity in their modes of reproduction: most species are reported to have either amphimixis or parthenogenesis, as well as less common modes such as autogamy or restitutional automixis (Pavlíček et al., 2023). Although it is generally considered that polyploidy in animals should be associated with parthenogenesis, there is no obvious association between these modes in earthworms: many polyploid races retain the ability to reproduce sexually. Furthermore, populations with sexual reproduction or partial degeneration of the sexual system were found in species that are considered parthenogenetic (Fernández et al., 2010). In other parthenogenetic species, there are genetic clues to possible sexual reproduction (Simonsen, Holmstrup, 2008). This flexibility in reproduction modes may contribute to the widespread occurrence of polyploidy in earthworms.

Some researchers suggested that polyploid races may have arisen as a result of allopolyploidization (Mezhzherin et al., 2018). However, it is important to note that none of the available molecular studies have yet provided evidence to support this hypothesis

## Conclusion

Based on the available data, we can conclude that the most frequent case in earthworms is “one genetic lineage – several ploidy races”, implying that this polyploidy is recent. However, in some instances, polyploid populations can survive for prolonged periods of time, giving rise to new genetic lineages.

## Conflict of interest

The authors declare no conflict of interest.

## References

Bakhtadze N.G., Kvavadze E.S., Bakhtadze G.I. Results of karyologic
investigation of Dendrobaena (C.) marinae Kvavadze, 1985 (Oligochaeta,
Lumbricidae). Bull. Georg. Natl. Acad. Sci. 2003;167(2):
315-316

Bakhtadze N., Bakhtadze G., Kvavadze E. The results of study of the
genus Dendrobaena (Oligochaeta, Lumbricidae) species chromosome
numbers. Bull. Georg. Acad. Sci. 2005;172(1):141-143

Blakemore R.J. Earthworms newly from Mongolia (Oligochaeta, Lumbricidae,
Eisenia). Zookeys. 2013;285:1-21. DOI 10.3897/zookeys.
285.4502

Bulatova N.S., Viktorov A.G., Perel T.S. Ecological heterogeneity of
polyploid forms of earthworms (Oligochaeta, Lumbricidae) with
special reference
to Eisenia nordenskioldi (Eisen). Proc. USSR
Acad. Sci. 1984;278:1020-1021 (in Russian)

Bulatova N., Perel T.S., Graphodatsky A.S. Constancy of the chromosome
set in polyploid earthworms with special reference to Eisenia
nordenskioldi (Oligochaeta, Lumbricidae). Bollettino di Zoologia.
1987;54(4):289-291. DOI 10.1080/11250008709355599

Casellato S. On polyploidy in Oligochaetes with particular reference to
Lumbricids. In: On Earthworms. Modena: Mucci, 1987;75-87

Casellato S., Rodighiero R. Karyology of Lumbricidae. III Contribution.
Caryologia. 1972;25(4):513-524. DOI 10.1080/00087114.1972.
10796504

Csuzdi C., Chang C.-H., Pavlícek T., Szederjesi T., Esopi D., Szlávecz
K. Molecular phylogeny and systematics of native North
American
lumbricid earthworms (Clitellata: Megadrili). PLoS One.
2017;12(8):e0181504. DOI 10.1371/journal.pone.0181504

Ermolov S.A., Shekhovtsov S.V., Geraskina A.P., Derzhinsky E.A.,
Kotsur V.M., Poluboyarova T.V., Peltek S.E. Morphological and genetic
analysis of Dendrodrilus rubidus (Bimastos rubidus) (Oligochaeta,
Lumbricidae) in Russia and Belarus. Russ. J. Ecosyst. Ecol.
2023;8(1):15-27. DOI 10.21685/2500-0578-2023-1-2

Fernández R., Novo M., Gutiérrez M., Almodóvar A., Díaz Cosín D.J.
Life cycle and reproductive traits of the earthworm Aporrectodea
trapezoides (Dugès, 1828) in laboratory cultures. Pedobiologia.
2010;53(5):295-299. DOI 10.1016/j.pedobi.2010.01.003

Fernández R., Novo M., Marchán D.F., Díaz Cosín D.J. Diversification
patterns in cosmopolitan earthworms: similar mode but different
tempo. Mol. Phylogenet. Evol. 2016;94(Pt. B):701-708. DOI
10.1016/j.ympev.2015.07.017

Giska I., Sechi P., Babik W. Deeply divergent sympatric mitochondrial
lineages of the earthworm Lumbricus rubellus are not reproductively
isolated. BMC Evol. Biol. 2015;15(1):217. DOI 10.1186/s12862-
015-0488-9

Golovanova E.V., Kniazev S.Y., Babiy K.A., Tsvirko E.I., Karaban K.,
Solomatin D.V. Dispersal of earthworms from the Rudny Altai
(Kazakhstan)
into Western Siberia. Ecol. Montenegrina. 2021;45:
48-61. DOI 10.37828/em.2021.45.9

Graphodatsky A.S., Perel T.S., Radzhabli S.I. Chromosome sets of two
forms of Eisenia nordenskioldi (Eisen) (Oligochaeta, Lumbricidae).
Doklady AN SSSR = Reports of the Academy of Sciences of USSR.
1982;262:1514-1516 (in Russian)

Gregory T.R., Mable B.K. Polyploidy in animals. In: The Evolution
of the Genome. San Diego: Elsevier, 2005;427-517. DOI 10.1016/
B978-012301463-4/50010-3

Heethoff M., Etzold K., Scheu S. Mitochondrial COII sequences indicate
that the parthenogenetic earthworm Octolasion tyrtaeum
(Savigny 1826) constitutes of two lineages differing in body size and
genotype. Pedobiologia. 2004;48(1):9-13. DOI 10.1016/j.pedobi.
2003.04.001

Holmstrup M., Simonsen V. Genetic and physiological differences
between two morphs of the lumbricid earthworm Dendrodrilus rubidus
(Savigny, 1826). Soil Biol. Biochem. 1996;28(8):1105-1107.
DOI 10.1016/0038-0717(96)00110-1

Hong Y., Csuzdi C. New data to the earthworm fauna of the Korean
Peninsula with redescription of Eisenia koreana (Zicsi) and remarks
on the Eisenia nordenskioldi species group (Oligochaeta, Lumbricidae).
Zool. Stud. 2016;55:12. DOI 10.6620/ZS.2016.55-12

Kashmenskaya M.N., Polyakov A.V. Karyotype analysis of five species
of earthworms (Oligochaeta: Lumbricidae). Comp. Cytogenet.
2008;2(2):121-125

King R.A., Tibble A.L., Symondson W.O.C. Opening a can of worms:
Unprecedented sympatric cryptic diversity within British lumbricid
earthworms. Mol. Ecol. 2008;17(21):4684-4698. DOI 10.1111/
j.1365-294X.2008.03931.x

Kodandaramaiah U. Tectonic calibrations in molecular dating. Curr.
Zool. 2011;57(1):116-124. DOI 10.1093/czoolo/57.1.116

Kvavadze E.Sh. The Earthworms (Lumbricidae) of the Caucasus. Tbilisi:
Metsniereba Publ., 1985 (in Russian)

Malevich I.I. On the study of earthworms in the Far East. Proc. V.P. Potemkin
MSPI. 1956;61(4/5):439-449 (in Russian)

Malinina T.V., Perel T.S. Characterization of Eisenia nordenskioldi
(Oligochaeta, Lumbricidae) chromosome races using allozyme
markers. Doklady Akademii Nauk SSSR = Reports of the Academy of
Sciences of USSR. 1984;279:1265-1269 (in Russian)

Marchán D.F., Cosín D.J.D., Novo M. Why are we blind to cryptic
species? Lessons from the eyeless. Eur. J. Soil Biol. 2018;86:49-51.
DOI 10.1016/j.ejsobi.2018.03.004

Martinsson S., Erséus C. Cryptic speciation and limited hybridization
within Lumbricus earthworms (Clitellata: Lumbricidae). Mol. Phylogenet.
Evol. 2017;106:18-27. DOI 10.1016/j.ympev.2016.09.011

Martinsson S., Erséus C. Hybridisation and species delimitation of
Scandinavian Eisenia spp. (Clitellata: Lumbricidae). Eur. J. Soil
Biol. 2018;88:41-47. DOI 10.1016/j.ejsobi.2018.06.003

Martinsson S., Rhodén C., Erséus C. Barcoding gap, but no support for
cryptic speciation in the earthworm Aporrectodea longa (Clitellata:
Lumbricidae). Mitochondrial DNA Part A. 2017;28(2):147-155.
DOI 10.3109/19401736.2015.1115487

Meinhardt U. Comparative observations on the laboratory biology of
endemic earthworm species. II. Biology of bred species. Zeitschrift
für Angew. Zool. 1974;61:137-182

Mezhzherin S.V., Garbar A.V., Vlasenko R.P., Onishchuk I.P., Kotsyuba
I.Y., Zhalai E.I. Evolutionary Paradox of the Parthenogenetic
Earthworms. Kiev: Naukova Dumka Publ., 2018 (in Russian)

Muldal S. The chromosomes of the earthworms: I. The evolution of
polyploidy. Heredity. 1952;6(1):56-76. DOI 10.1038/hdy.1952.4

Muller H.J. Why polyploidy is rarer in animals than in plants. Am. Nat.
1925;59(663):346-353. DOI 10.1086/280047

Omodeo P. Cariologia dei Lumbricidae: (con Tavole XIV–XV e 27
figure nel testo). Caryologia. 1952;4(2):173-275. DOI 10.1080/
00087114.1952.10797539

Omodeo P. Cariologia dei Lumbricidae II. Contributo (con 8 tabelle
e 16 figure nel testo). Caryologia. 1955;8(1):135-178. DOI 10.1080/
00087114.1955.10797555

Orr H.A. “Why polyploidy is rarer in animals than in plants” revisited.
Am. Nat. 1990;136(6):759-770. DOI 10.1086/285130

Pavlíček T., Szederjesi T., Pearlson O., Csuzdi C. Biodiversity and distribution
of earthworms in the biogeographic province of the Levant.
Zool. Middle East. 2023;69(4):394-409. DOI 10.1080/09397140.
2023.2279360

Perel T.S. Earthworms in forest soils of the Northwestern Caucasus. In:
The Impact of Animals on the Productivity of Forest Biogeocenoses.
Moscow: Nauka Publ., 146-165 (in Russian)

Perel T.S., Graphodatsky A.S. New species of the genus Eisenia (Lumbricidae,
Oligochaeta) and their chromosome sets. Zoologicheskiy
Zhurnal. 1984;63(4):610-612

Porco D., Decaëns T., Deharveng L., James S.W., Skarzyński D., Erséus
C., Butt K.R., Richard B., Hebert P.D.N. Biological invasions in
soil: DNA barcoding as a monitoring tool in a multiple taxa survey
targeting European earthworms and springtails in North America.
Biol. Invasions. 2013;15(4):899-910. DOI 10.1007/s10530-012-
0338-2

Ronquist F., Teslenko M., Van Der Mark P., Ayres D.L., Darling A.,
Höhna S., Larget B., Liu L., Suchard M.A., Huelsenbeck J.P.
MrBayes 3.2: efficient Bayesian phylogenetic inference and model
choice across a large model space. Syst. Biol. 2012;61(3):539-542.
DOI 10.1093/sysbio/sys029

Shekhovtsov S.V., Golovanova E.V., Peltek S.E. Cryptic diversity
within the Nordenskiold’s earthworm, Eisenia nordenskioldi subsp.
nordenskioldi (Lumbricidae, Annelida). Eur. J. Soil Biol. 2013;58:
13-18. DOI 10.1016/j.ejsobi.2013.05.004

Shekhovtsov S.V., Golovanova E.V., Peltek S.E. Genetic diversity of
the earthworm Octolasion tyrtaeum (Lumbricidae, Annelida). Pedobiologia.
2014;57(4-6):245-250. DOI 10.1016/j.pedobi.2014.09.002

Shekhovtsov S.V., Berman D.I., Bazarova N.E., Bulakhova N.A., Porco
D., Peltek S.E. Cryptic genetic lineages in Eisenia nordenskioldi
pallida (Oligochaeta, Lumbricidae). Eur. J. Soil Biol. 2016;75:151-
156. DOI 10.1016/j.ejsobi.2016.06.004

Shekhovtsov S.V., Berman D.I., Bulakhova N.A., Vinokurov N.N.,
Peltek S.E. Phylogeography of Eisenia nordenskioldi nordenskioldi
(Lumbricidae, Oligochaeta) from the north of Asia. Polar Biol.
2018;41(2):237-247. DOI 10.1007/s00300-017-2184-2

Shekhovtsov S.V., Ershov N.I., Vasiliev G.V., Peltek S.E. Transcriptomic
analysis confirms differences among nuclear genomes of cryptic
earthworm lineages living in sympatry. BMC Evol. Biol. 2019;
19(Suppl. 1):50. DOI 10.1186/s12862-019-1370-y

Shekhovtsov S.V., Derzhinsky Y.A., Poluboyarova T.V., Golovanova
E.V., Peltek S.E. Phylogeography and genetic lineages of Aporrectodea
rosea (Lumbricidae, Annelida). Eur. J. Soil Biol. 2020a;99:
103191. DOI 10.1016/j.ejsobi.2020.103191

Shekhovtsov S.V., Ermolov S.A., Derzhinsky Ye.A., Poluboyarova
T.V., Laricheva M.S., Peltek S.E. Genetic and body size variation
in Octolasion tyrtaeum (Lumbricidae, Annelida). Pisma v Vavilovskii
Zhurnal Genetiki i Selektsii = Letters to Vavilov Journal of Genetics
and Breeding. 2020b;6(1):5-9. DOI 10.18699/Letters2020-6-01 (in
Russian)

Shekhovtsov S.V., Rapoport I.B., Poluboyarova T.V., Geraskina A.P.,
Golovanova E.V., Peltek S.E. Morphotypes and genetic diversity of
Dendrobaena schmidti (Lumbricidae, Annelida). Vavilovskii Zhurnal
Genetiki i Selektsii = Vavilov Journal of Genetics and Breeding.
2020c;24(1):48-54. DOI 10.18699/VJ20.594

Shekhovtsov S.V., Shipova A.A., Poluboyarova T.V., Vasiliev G.V.,
Golovanova E.V., Geraskina A.P., Bulakhova N.A., Szederjesi T.,
Peltek S.E. Species delimitation of the Eisenia nordenskioldi complex
(Oligochaeta, Lumbricidae) using transcriptomic data. Front.
Genet. 2020d;11:1508. DOI 10.3389/fgene.2020.598196

Shekhovtsov S.V., Efremov Y.R., Poluboyarova T.V., Peltek S.E. Variation
in nuclear genome size within the Eisenia nordenskioldi complex
(Lumbricidae, Annelida). Vavilovskii Zhurnal Genetiki i Selektsii
= Vavilov Journal of Genetics and Breeding. 2021;25(6):647-651.
DOI 10.18699/VJ21.073

Shekhovtsov S.V., Shipova A.A., Bulakhova N.A., Berman D.I. Differentiation
within the Drawida ghilarovi complex (Moniligastridae:
Annelida) revealed by multigene transcriptomic dataset analysis.
Eur. J. Soil Biol. 2022;111:103411. DOI 10.1016/j.ejsobi.2022.
103411

Simonsen V., Holmstrup M. Deviation from apomictic reproduction
in Dendrobaena octaedra? Hereditas. 2008;145(4):212-214. DOI
10.1111/j.0018-0661.2008.02045.x

Sims R.W., Gerard B.M. Earthworms: Notes for the Identification of
British Species. London, 1999

Stamatakis A. RAxML version 8: a tool for phylogenetic analysis and
post-analysis of large phylogenies. Bioinformatics. 2014;30(9):
1312-1313. DOI 10.1093/bioinformatics/btu033

Taheri S., James S., Roy V., Decaëns T., Williams B.W., Anderson F.,
Rougerie R., Chang C.-H., Brown G., Cunha L., Stanton D.W.G.,
Da Silva E., Chen J.-H., Lemmon A.R., Moriarty Lemmon E.,
Bartz M., Baretta D., Barois I., Lapied E., Coulis M., Dupont L.
Complex taxonomy of the ‘brush tail’ peregrine earthworm Pontoscolex
corethrurus. Mol. Phylogenet. Evol. 2018;124:60-70. DOI
10.1016/j.ympev.2018.02.021

Vedovini A. Systématique, caryologie et écologie des oligochètes
terrestres de la région provençale. Centre de documentation du
C.N.R.S. Thèse. Fac. Sci. Univ. Provence, 1973

Viktorov A.G. Ecology, Caryology, and Radiosensitivity of Earthworm
Races with Different Ploidy. Moscow, 1989 (in Russian)

Viktorov A.G. Polyploid race variety in the earthworms family Lumbricidae.
Uspekhi Sovremennoy Biologii = Biology Bulletin Reviews.
1993;113(3):304-312 (in Russian)

Viktorov A.G. Diversity of polyploid races in the family Lumbricidae.
Soil Biol. Biochem. 1997;29(3-4):217-221. DOI 10.1016/S0038-
0717(96)00086-7

Vlasenko R.P., Mezhzherin S.V., Garbar A.V., Kotsuba Y. Polyploid
races, genetic structure and morphological features of earthworm
Aporrectodea rosea (Savigny, 1826) (Oligochaeta, Lumbricidae) in
Ukraine. Comp. Cytogenet. 2011;5(2):91. DOI 10.3897/compcytogen.
v5i2.968

Vsevolodova-Perel T.S. The Earthworms of the Russian Fauna: Cadaster
and Key. Moscow: Nauka Publ., 1997 (in Russian)

Vsevolodova-Perel T.S. Addition to the fauna of earthworms (Oligochaeta,
Lumbricidae) of the Northern Caucasus. Zoologicheskiy
Zhurnal. 2003;82(2):275-280 (in Russian)

Vsevolodova-Perel T.S., Bulatova N.S. Polyploid races of earthworms
(Lumbricidae, Oligochaeta) in the East European plain and Siberia.
Biol. Bull. Russ. Acad. Sci. 2008;35(4):385-388. DOI 10.1134/
S1062359008040092

Vsevolodova-Perel T.S., Leirikh A.N. Distribution and ecology of
the earthworm Eisenia nordenskioldi pallida (Oligochaeta, Lumbricidae)
dominant in Southern Siberia and the Russian Far East.
Zoologicheskiy Zhurnal. 2014;93(1):45-52. DOI 10.7868/S00445
13414010206 (in Russian)

